# *TakeTwo*: an indexing algorithm suited to still images with known crystal parameters

**DOI:** 10.1107/S2059798316010706

**Published:** 2016-07-28

**Authors:** Helen Mary Ginn, Philip Roedig, Anling Kuo, Gwyndaf Evans, Nicholas K. Sauter, Oliver Ernst, Alke Meents, Henrike Mueller-Werkmeister, R. J. Dwayne Miller, David Ian Stuart

**Affiliations:** a Division of Structural Biology, Wellcome Trust Centre for Human Genetics, Roosevelt Drive, Oxford OX3 7BN, England; b Diamond House, Harwell Science and Innovation Campus, Fermi Avenue, Didcot OX11 0QX, England; c Deutsches Elektronen-Synchrotron, Notkestrasse 85, 22607 Hamburg, Germany; d Department of Biochemistry, University of Toronto, King’s College Circle, Toronto, ON M5S 1A8, Canada; e Molecular Biophysics and Integrated Bioimaging Division, Lawrence Berkeley National Laboratory, 1 Cyclotron Road, Berkeley, CA 94720, USA; f Department of Molecular Genetics, University of Toronto, King’s College Circle, Toronto, ON M5S 1A8, Canada; g Atomically Resolved Dynamics, Max-Planck-Institute for Structure and Dynamics of Matter, Luruper Chaussee 149, Hamburg, Germany; h Hamburg Centre for Ultrafast Imaging, University of Hamburg, Hamburg, Germany; i Departments of Physics and Chemistry, University of Toronto, 80 St George Street, Toronto, ON M5S 1H6, Canada

**Keywords:** *TakeTwo*, data processing, serial crystallography, XFELs, X-ray free-electron lasers

## Abstract

The indexing methods currently used for serial femtosecond crystallography were originally developed for experiments in which crystals are rotated in the X-ray beam, providing significant three-dimensional information. On the other hand, shots from both X-ray free-electron lasers and serial synchrotron crystallo­graphy experiments are still images, in which the few three-dimensional data available arise only from the curvature of the Ewald sphere. Traditional synchrotron crystallography methods are thus less well suited to still image data processing. Here, a new indexing method is presented with the aim of maximizing information use from a still image given the known unit-cell dimensions and space group. Efficacy for cubic, hexagonal and orthorhombic space groups is shown, and for those showing some evidence of diffraction the indexing rate ranged from 90% (hexagonal space group) to 151% (cubic space group). Here, the indexing rate refers to the number of lattices indexed per image.

## Abstract 2

A novel indexing method is presented that is well suited to the minimal information on a still image diffraction pattern and can achieve indexing rates of over one lattice per image.

## 1. Introduction   

Indexing, or deducing the specimen orientation from crystalline diffraction patterns, can potentially be performed with high accuracy and precision owing to the integral nature of the Miller indices at which Bragg reflections are located. Indexing algorithms implemented in programs such as *XDS* (Kabsch, 1993 ▸), *iMosflm* (Powell *et al.*, 2013 ▸), *DENZO* (Otwinowski & Minor, 2006 ▸), *LABELIT* (Sauter *et al.*, 2004 ▸) and *DIALS* (Gildea *et al.*, 2014 ▸) are well established for data-collection strategies that involve crystal rotation, which are typically employed at synchrotron sources. For data collected at X-ray free-electron laser (XFEL) sources, where each image represents diffraction from a separate nonrotating specimen, the measure of success is less well defined. Data-analysis pipelines sometimes include a preprocessing ‘hitfinder’ step, which distinguishes images without diffraction (blanks) from hits that exceed a certain threshold number of candidate Bragg spots, which is typically set to around 20. The indexing rate is therefore defined as the percentage of hits for which crystal orientations can be determined. Reported indexing rates are often quite low (Barends *et al.*, 2013 ▸; Liu *et al.*, 2013 ▸; Johansson *et al.*, 2013 ▸; Ginn, Brewster *et al.*, 2015 ▸; Chapman *et al.*, 2011 ▸), although the rate depends on a number of factors, including the strength of diffraction on each image and the diffraction resolution. Our own two analyses of diffraction data from Cypovirus type 17 polyhedrin (CPV17) yielded mediocre indexing rates of 53% (Ginn, Messerschidt *et al.*, 2015 ▸) and 36% (Ginn, Brewster *et al.*, 2015 ▸). Here, we investigate whether alternate algorithms can help improve the indexing rate, with the overall goal of producing good-quality structures while consuming a minimal amount of crystalline sample and beam time.

All indexing methods, regardless of the data source, begin by transforming candidate Bragg spot coordinates measured on the detector into corresponding three-dimensional coordinates in reciprocal space. The periodic arrangement of reciprocal-lattice points (rlps) is then detected using one of several methods. For rotation data generally, equivalent determinations of the three-dimensional periodic repeat can be deduced either by a single three-dimensional fast Fourier transformation of the entire reciprocal-space pattern (Campbell, 1998 ▸) or by separately computing one-dimensional FFTs of the pattern projected onto individual directional axes in reciprocal space and considering all possible directional axes, finely sampled on a spherical grid (Steller *et al.*, 1997 ▸). A key success factor is that the specimen rotation provides sufficient sampling in three dimensions to dramatically overdetermine the three-dimensional lattice repeat. In contrast, still shots produced at XFEL sources contain only the limited three-dimensional information that is afforded by the curvature of the Ewald sphere, which becomes essentially non-existent in the low-resolution limit. Another condition making it difficult to detect periodicity from still shots is the minimal number of Bragg spots that meet the reflection conditions, especially from crystals with smaller unit-cell dimensions.

A number of approaches have been adopted to mitigate these inherent difficulties in resolving the lattice for still-shot data. The *cctbx.xfel* software suite (Hattne *et al.*, 2014 ▸) uses the one-dimensional FFT method to identify candidate basis vectors that can potentially span the lattice, but then uses prior knowledge of the unit-cell parameters to choose a basis set (three vectors combined) that best agrees with the known unit-cell lengths and angles. Even if the unit cell is initially unknown, a good target cell may be derived from an initial first pass of data reduction (Zeldin *et al.*, 2015 ▸). Secondly, a recent method implemented within the *DIALS* toolbox (Waterman *et al.*, 2013 ▸) avoids the one-dimensional FFT search altogether (Gildea *et al.*, 2014 ▸). The basic idea here is that the FFT is primarily useful to identify the periodic repeat spacing if the unit-cell length is completely unknown. However, since the typical unit-cell length can be treated as prior knowledge, it is sufficient to perform an exhaustive search for the already known periodic spacings over a grid of directional axes. This method has a high success rate for finding the basis vectors from XFEL still shots, and has also been used to index shots where multiple lattices are present, including CPV polyhedrin diffraction data from Diamond Light Source beamline I24, which eventually resulted in structure solution (Ginn, Messerschmidt *et al.*, 2015 ▸). A final adaptation concerns the rocking curve that is normally observed in rotation data sets, as the reciprocal-lattice point is first rotated into and then out of the exact reflecting condition. At synchrotrons it is easy to collect individual image frames with a fine enough rotational slicing to determine the spot centroid position with high accuracy, but for still data the degree to which the rlps are offset from the Ewald sphere is initially unknown. To compensate, parameter refinement targets have been developed (Sauter *et al.*, 2014 ▸; Kabsch, 2014 ▸) that restrain the lattice orientation (after the basis vectors are chosen) so that the rlps are positioned as close as possible to the Ewald sphere.

In our efforts to analyse a number of new challenging data sets, including that of *Bovine enterovirus* (BEV), we revisited the indexing stage of the data-processing pipeline. At first, the BEV data appeared to be intractable owing to the low signal to noise and small separation between neighbouring spots. Initial attempts at indexing, specifying the known unit cell and space group, produced indexing solutions with refinement statistics that appeared to be of extremely good quality. Finally, however, we realised that the solutions were fundamentally incorrect owing to a large (20 mm) error in the detector distance that initially went undetected.

In the process of developing automated approaches to fix the distance problem, we also sought new ways to detect the basis vectors, considering the sparsity of Bragg spots that potentially precludes the use of FFT methods. Older indexing methods developed around 1990 (Kabsch, 1988 ▸; Higashi, 1990 ▸; Kabsch, 1993 ▸) succeeded without any explicit grid search or FFT method by considering the difference vectors that connect rlps that are close in reciprocal space. Kabsch (1988 ▸) was able to index rotation data by creating a three-dimensional histogram of such difference vectors and identifying clusters which correspond to candidate basis vectors for the lattice.

Inspired by these early experiences with indexing from a limited set of difference vectors, we devised a similar algorithm that allows us to tackle difficult diffraction patterns and correct for errors in the measurements of the experimental geometry. We have named this algorithm *TakeTwo*, reflecting the underlying idea of taking pairs of spots to form vectors and pairs of vectors to generate indexing solutions. *TakeTwo* attempts to make maximal use of the information contained within a single still image by considering all inter-spot vectors that could match to a vector between reflections in reciprocal space. This indexing algorithm provides high indexing rates for cubic and hexagonal space groups, and a high degree of success in indexing multiple lattices. *TakeTwo* is applicable to both serial synchrotron and serial femtosecond crystallo­graphy. We expect the *TakeTwo* algorithm to markedly improve the indexing rates of many of the available XFEL data sets.

## 2. Materials and methods   

### 2.1. Data acquisition for various protein crystals   

A summary of the experimental parameters and crystal symmetry for the various data sets is provided in Table 1 ▸. The diffraction patterns used for CPV17 had previously been used for structure determination (Ginn, Brewster *et al.*, 2015 ▸; Ginn, Messerschmidt *et al.*, 2015 ▸). Data were collected at the XPP endstation at the Linac Coherent Light Source (LCLS) under proposal LH90 from BEV crystals on a silicon chip (Roedig *et al.*, 2015 ▸; cubic space group *F*23, unit-cell dimension 437 Å). Reflections were observed to approximately 2.0 Å resolution and the minimum separation of individual reflections was three pixels. The data from thermolysin crystals, collected in 2011, have previously been processed and optimal unit-cell dimensions established (Uervirojnangkoorn *et al.*, 2015 ▸). All of the above data were recorded using the CSPAD detector. Diffraction patterns from myoglobin crystals crystallized in space group *P*2_1_2_1_2_1_ provided an example of an orthorhombic space group and were recorded using a Rayonix MX170-HS detector at the XPP endstation using an alternative silicon-chip delivery system (Sherrell *et al.*, 2015 ▸; Zarrine-Afsar *et al.*, 2012 ▸; Mueller *et al.*, 2015 ▸), which has been shown to support structure solution (Oghbaey *et al.*, 2016 ▸).

### 2.2. Hit finding and spot finding using *DIALS*   

Hits for the CPV17, BEV and thermolysin data sets were determined with *cctbx.xfel* using the default minimum spot count of 20 to distinguish a hit from a non-useful image. Images for the myoglobin data set were identified as hits if they had at least 30 spots from *DIALS* (Waterman *et al.*, 2013 ▸) spot-finding analysis, which uses *XDS* algorithms (Kabsch, 1977 ▸). For our indexing algorithm, spots were identified using *DIALS*, manually finding an optimal combination of spot-finding parameters (Table 2 ▸). *DIALS* produced spot-centroid coordinates on the surface of the detector.

### 2.3. Generating inter-spot vectors   

Spots were back-projected onto the Ewald sphere using the known experimental parameters (incident energy and detector geometry). Three-dimensional vectors were calculated between spots in reciprocal space. The coordinates and vectors generated from back-projection onto the Ewald sphere are referred to as ‘observed space’, as they come directly from the spots and parameters of the experiment.

### 2.4. Optional filtering of inter-spot vectors   

In cases where substantial amounts of noise are picked up by the spot-finding algorithms or multiple lattices are found on the same image, one can perform an optional filtering of inter-spot vectors to help prevent spot-to-noise, noise-to-noise or inter-lattice vectors from being included in pseudo-powder pattern generation or indexing. This was performed by tabularing all inter-spot vectors and removing those which had the fewest neighbours, as explained below.

An inter-spot vector was considered to be a neighbour of another vector if they were within a certain tolerance of each other. The tolerance was either calculated for a given resolution based on the energy bandwidth and the rlp size, or alternatively a constant tolerance was assumed across the entire image. The variable distance tolerance was chosen as the maximum possible separation between rlps of finite size on opposite sides of the nest of Ewald spheres. This assures that the vector is of a similar length and direction, and is more likely to be a repeated vector from the same lattice. Each vector was assigned a score which was equal to the total number of neighbouring vectors. The vectors were ordered in terms of their score in descending order, and a particular fraction of the vectors were removed if they did not reach a threshold *p*_*t*_, which was based on the number of expected lattices for a given image, as follows. The number of expected lattices (*n*) on the image was calculated based on the number of spots on the image (*s*) divided by the number expected per lattice (*l*). The value of *l* was manually estimated after obtaining a few indexing results as the average number of spots picked by *DIALS* which were predicted (and removed) by one lattice solution. *n* was rounded to the nearest integer: 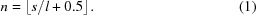
The total number of inter-spot vectors for a single lattice is proportional to the square of the total number of spots. However, if there were two lattices on an image, each spot could generate a vector either with a member of its own lattice or the other lattice, with roughly equal probability. Thus, one would only expect half of the spot vectors to originate between member vectors of the same lattice. The proportion of vectors (*p*_*t*_) which were likely to originate from a single lattice can therefore be determined: 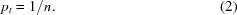
The threshold *t* was set so that only the proportion *p*_*t*_ of vectors would remain. Since the list is ordered so that those with the most neighbours are at the top, taking this proportion from the top of the list aims to enrich those that originate from the same lattice.

### 2.5. Theoretical distances for crystal lattices and space groups   

Theoretical distances between individual reflections according to the space group and unit-cell dimension were calculated by considering all Miller indices and their relation to the origin of reciprocal space. Care was taken over systematic absences: those owing to the centring type of the lattice of the crystal prevent some inter-spot distances from ever appearing, as opposed to axial systematic absences which do not affect the occurrence of general inter-spot distances. The reciprocal unit cell generated by applying the transformation matrix onto Cartesian coordinates is hereby referred to as ‘theoretical space’. The indexing solution then defines the orientation matrix which was applied to the transformation matrix to give rise to the spots observed on the detector.

### 2.6. One-dimensional pseudo-powder patterns   

**Figure 1:**
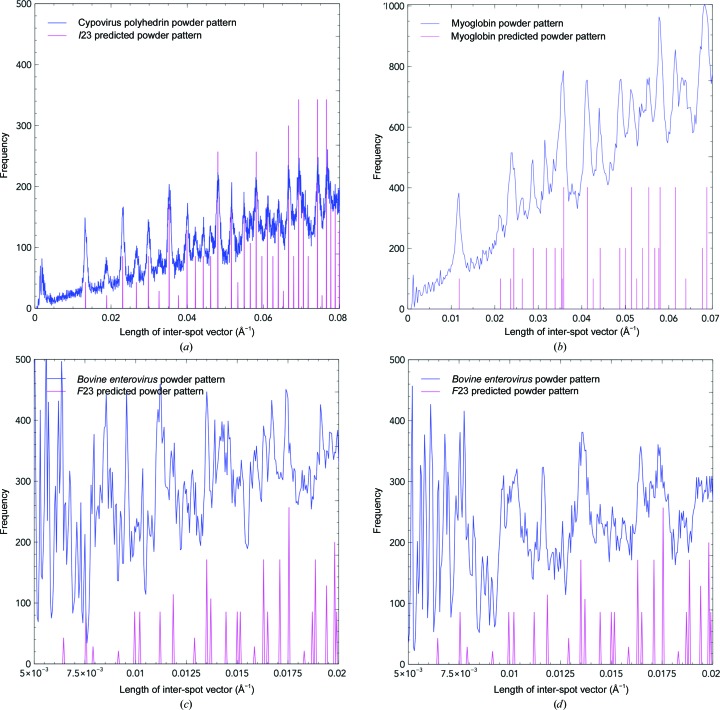
CPV17 at 101.2 mm detector distance (*a*), myoglobin (*b*) and BEV pseudo-powder patterns (*c*, *d*) generated using optimized experimental parameters without spot-vector filtering. The blue line is generated from experimental data, whereas the pink line is the expected pseudo-powder pattern given the unit-cell and space-group information. The height of the magenta line denotes the relative number of Miller indices which give rise to the particular inter-spot vector length; the absolute values are not shown for clarity. The CPV17 powder pattern was generated from 262 images. Owing to the higher mosaicity of the myoglobin crystals (464 images), the experimental peaks for each powder ring in (*b*) are broadened compared with the CPV17 powder pattern (*a*). For the BEV pseudo-powder patterns (304 images), the pattern in (*c*) was generated from the recorded detector distance of 105 mm. However, the pattern in (*d*), which shows a better fit to the pseudo-powder pattern, was obtained at a detector distance of 85 mm. The peaks are less well separated owing to the high error associated with measuring spot vectors between extremely close spots.

One-dimensional pseudo-powder patterns were generated from a small number of diffraction patterns (Fig. 1 ▸) by generating a histogram of inter-spot distances. These were compared with the predicted reflections given the unit-cell dimensions and space-group systematic absences, and the wavelength and detector distance were adjusted to ensure a good fit between the theoretical and observed powder diffraction patterns. The beam centre correctness, metrology, detector distance and wavelength parameters all affected the sharpness of the powder rings.

### 2.7. Inter-spot vector-distance matching   

In order to use the observed space vectors for indexing, they were filtered according to length. Observed space vectors are considered for indexing if they match one or more of the theoretical space vectors in length, within a certain tolerance, calculated as in §[Sec sec2.4]2.4.

### 2.8. First indexing method: rotation-matrix clusters   

Two related indexing algorithms based on inter-spot vectors have been created which are suited to different situations. The *TakeTwo* algorithm therefore has two branches: the rotation-matrix cluster method and the inter-spot vector-network method.

An indexing solution cannot be determined by one inter-spot vector alone as this only anchors reciprocal space in one axis. An indexing solution must be generated by a minimum of two vectors which are not linearly dependent. This mostly used four spots, but can use three if one spot is shared between the two inter-spot vectors, as explained in §[Sec sec2.10]2.10. Inter-spot vectors were considered a candidate for a solution if the angle between them in the observed space matched the angle between matching vectors in the theoretical space within 1°. For each of these pairs of inter-spot vectors, a matrix was generated which aligns the observed space onto the theoretical space (the opposite transformation was achieved using the inverse). This was combined with the transformation matrix to generate a potential indexing solution. Owing to the presence of noise and multiple lattices on a single image, the map of orientation matrices produced using this method had a low signal-to-noise ratio. Indexing solutions were chosen by selecting matrices which were closely surrounded by neighbouring solutions, determined by a reimplementation of the matrix similarity metric contained within *X-PLOR* (Brünger, 1990 ▸, 1992 ▸), which is reproduced here for clarity. For two rotation matrices *P* and *Q*, and *n* symmetry operators where *O_s_* is the rotational component of symmetry operator *s*, the metric 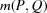 is defined as 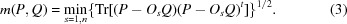
If this metric has a value below the threshold of 0.25, this corresponds to a rotation of 10° or less and the two are therefore treated as duplicate solutions.

To visualize the indexing solutions generated using this method, a standard unit vector, for example (1, 0, 0), was rotated by an orientation matrix to generate a new vector. This was decomposed into polar coordinates and the two angles (θ, φ) were plotted. Of course, this did not contain all of the information contained within the orientation matrices, but provides a two-dimensional representation which was easily plotted to visually identify clusters of orientation matrices (Fig. 3). Clusters were identified by having the highest number of neighbours (calculated using the *X-PLOR* method) within an 8° radius between the central spot and any potential neighbour. Indexing solutions were filtered such that symmetrically identical solutions did not occur more than once, allowing the selection of multiple lattices. Indexing solutions were then selected for initial orientation-matrix refinement according to a previously outlined protocol (Ginn, Messerschmidt *et al.*, 2015 ▸).

Indexing solutions were accepted on the basis of the success of the initial orientation-matrix refinement. These initial refinements generate histograms of Ewald sphere wavelengths, which are defined as the inverse of the Ewald sphere radius on which the centre of the modelled rlp lies. Images were accepted if their standard deviation of rlp midpoint Ewald sphere radii were below a certain threshold σ_*t*_ or if the histogram of wavelength peaks had a sufficiently high peak. These thresholds were manually chosen on a case-by-case basis for each crystal form and are highly dependent on the other parameters of the initial orientation-matrix refinement.

### 2.9. Second indexing method: building up an inter-spot vector network   

The second indexing method built up an interconnected network of vectors which were all self-consistent for a single indexing solution. The network was considered as an indexing solution if the number of vectors reached a certain threshold, to distinguish it from ‘noise’. This threshold was set to 20 by default. An indexing solution was built up recursively from a starting vector by adding further vectors which belong to the same indexing solution. In most cases, a new vector was not added unless it shared at least one spot with the existing network to increase the likelihood of picking spots from only a single lattice. However, this could be toggled in cases where only single lattices were suspected to be present. In order to increase the speed of calculation, vectors were ‘pre-screened’ to ensure that the angles between the pre-screened vector and the current network of existing vectors were consistent. The resulting orientation matrices between the pre-screened vector and existing vectors were then calculated according to §[Sec sec2.10]2.10 and the vector was accepted if it matched within a 8° tolerance of the existing indexing solutions as judged by the *X-PLOR* metric.

### 2.10. Generating an orientation matrix from two identified inter-spot vectors   

A rotation matrix was generated from two identified inter-spot vectors *i* and *j*. Reciprocal space was rotated to line up the first observed vector with the corresponding theoretical vector. The cross product and angle between the observed vector *i*_obs_ and the theoretical vector *i*_thr_ were calculated:
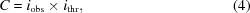


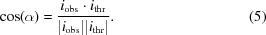
We then generated a rotation matrix **Q**_*a*_, rotating by the angle α around the axis *C*, which aligns one axis in reciprocal space. The second axis must be aligned using the second vector *j*_obs_. The rotation matrix **Q**_*b*_ rotates *j*_obs_ by an angle β around the axis *i*_thr_ such that the angle between **Q**_*b*_·*j*_obs_ and *j*_thr_ is minimized. Let **Q**_*c*_ be **Q**_*b*_ **Q**_*a*_. The inverse of **Q**_*c*_, **Q**_*c*_^−1^, equals **Q**_*c*_^T^, and is now equivalent to the **U** matrix defined by Busing & Levy (1967 ▸). The unit-cell transformation matrix is generated from the unit-cell parameters, which is equivalent to the Busing–Levy **B** matrix. The entire orientation matrix **R** is calculated as
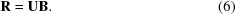
This matrix *R* can be applied to integer (*h*, *k*, *l*) values to map them onto rotated Cartesian coordinates in reciprocal space.

### 2.11. Indexing additional lattices using the inter-spot vector network   

The indexing of additional lattices using the inter-spot vector network is made possible by a previous method (Gildea *et al.*, 2014 ▸) in which spots which are predicted by an existing solution are removed from the data set and indexing is resumed with the remaining spots. Inter-spot vector networks which lead to a previously determined orientation matrix, or a geometrically equivalent matrix, are rejected. Solutions are scored for their equivalence as described above.

## 3. Results   

### 3.1. One-dimensional pseudo-powder patterns   

We find that well defined pseudo-powder patterns (generated as described in §[Sec sec2]2) can be obtained even when true powder patterns cannot be usefully calculated. For instance, when few patterns are available pseudo-powder patterns can still be produced. These are not only useful for diffraction patterns with small unit-cell dimensions (Fig. 1*a* ▸), but are still interpretable for diffraction patterns from crystals with large unit cells and very poor reflection separation, as for BEV crystals (Fig. 1 ▸ *c*).

**Figure 2:**
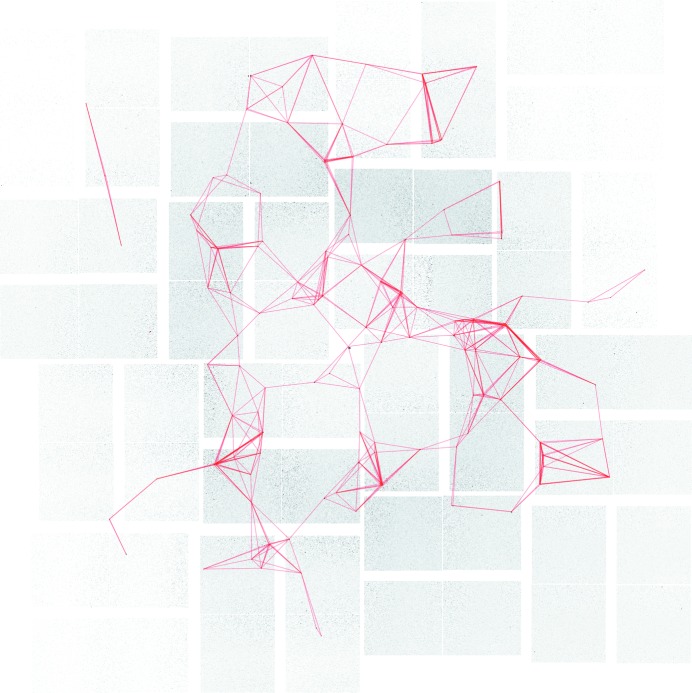
An example CPV17 crystal diffraction image at 101.2 mm detector distance with vectors picked between spots which lie within 0.1 Å^−1^ of each other. This will contribute a small portion of the pseudo-powder pattern as seen in Fig. 1 ▸. The Miller index (0, 0, 0) is also included in the analysis to boost the number of inter-spot vectors identified within the image.

An example of the vectors used in a single image to contribute to the overall pseudo-powder pattern is shown for CPV17 crystals in Fig. 2 ▸. The conventional powder patterns generated by overlaying multiple images may show fewer peaks than the pseudo-powder patterns owing to axial systematic absences. The corresponding distances will not be present in a conventional powder pattern, but will appear in the pseudo-powder pattern as such vectors occur off-axis.

As the number of BEV crystals was very small (304 images), a conventional powder pattern could not be generated by superimposition of the images, and if it had been possible, the rings would be impossible to distinguish: the unit-cell dimension is 437 Å and even the longest reciprocal-space distance in the pseudo-powder pattern (50 Å) only spanned 8 mm across the detector and was therefore lost in the unrecorded low-angle region. Only after a one-dimensional pseudo-powder pattern had been generated was it revealed that the original detector-distance reading was misaligned by 20 mm. This value could be refined manually by observing the effect on the pseudo-powder pattern (Figs. 1 ▸ *c* and 1 ▸ *d*). This enabled indexing of these diffraction patterns in a situation where the presence of this anomaly was otherwise difficult to establish. The BEV images had very poor separation of individual reflections, with the Miller index vector (1, 1, 1) corresponding to only three pixels. Assuming an error in a spot position of ∼0.5 pixels, the error in such a vector will be about 23%, having a major impact on the pseudo-powder pattern especially at small reciprocal distance values.

Owing to the orthorhombic nature of myoglobin, powder rings will overlap in ways which are not observed in cubic space groups. This will lead to a degree of uncertainty during the indexing stage when assigning theoretical vectors to observed vectors according to their distances. The pseudo-powder pattern for the myoglobin data set is shown in Fig. 1 ▸(*b*).

### 3.2. Rotation-matrix clusters   

**Figure 3:**
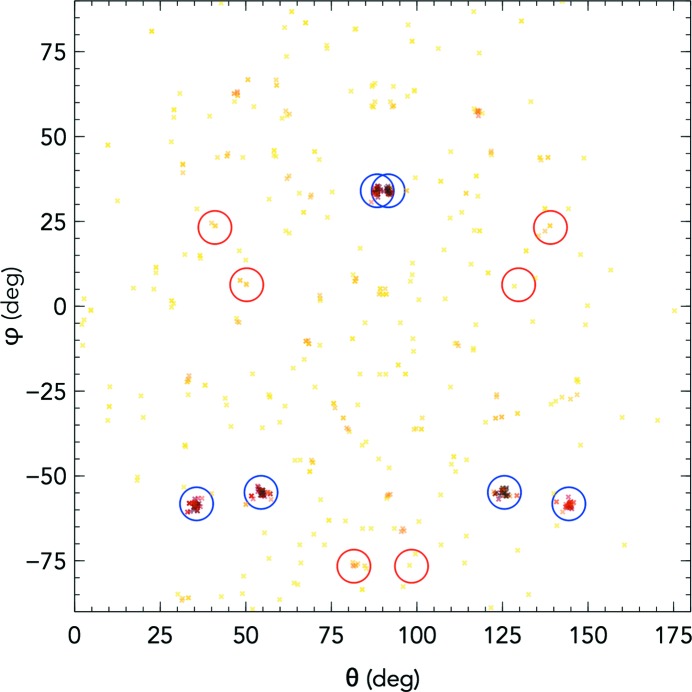
Matrix-cluster solutions decomposed into two dimensions, showing one strong (denoted by blue circles) and one weak solution (denoted by red circles) on the same image. Darker areas show matrices which have a high number of neighbouring solutions within an 8° angle, whereas yellow solutions have the lowest number of neighbours within the 8° angle and are likely to be noise. Both circles correspond to solutions which lead to a successful, unique indexing solution. Each solution has six symmetry-related solutions generated by indexing relative to geometrically equivalent axes. The image is from a CPV17 sample at 101.2 mm.

**Figure 4:**
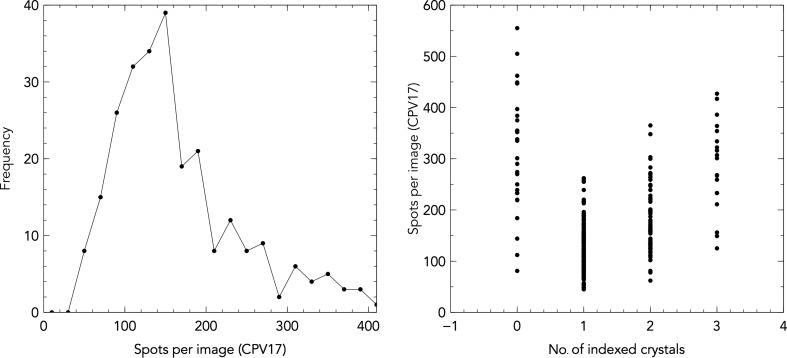
Left: a histogram of frequency of images with a given number of spots for the CPV17 sample at 101.2 mm detector distance. Images with higher numbers of spots typically have larger numbers of lattices. The graph is cropped at a maximum of 400 spots for clarity. Right: the frequency of spots on the same images grouped by the number of successfully indexed lattices. Images which fail to index typically have significantly larger spot counts.

The rotation-matrix cluster method, as described in §[Sec sec2]2, has a poor signal-to-noise ratio and can only reliably reach above the level of noise in cubic space groups, where only geo­metrically equivalent powder rings overlap. Projecting the orientation matrices onto two dimensions shows clusters of orientation matrices which have a high number of neighbours, and these clusters lead to indexing solutions (Fig. 3 ▸). However, this method is not well suited to the hexagonal space group of thermolysin, perhaps because of the less distinct separation of powder rings. A sample of 262 CPV17 images demonstrated a 130% indexing rate (*i.e.* more than one lattice per hit). Of all the images, 87% provided at least one lattice, with data extending to 1.98 Å at the edge of the detector. These images often contained multiple lattices, which were identifiable by the abnormally high numbers of spots (Fig. 4 ▸). The indexing rates obtained for these images in the previously published structure (Ginn, Messerschmidt *et al.*, 2015 ▸) was 52%.

### 3.3. Inter-spot vector networks   

Inter-spot vector networks were successfully applied to all of the test systems. For CPV17, inter-spot vector distances were considered up to a reciprocal distance of 0.19 Å^−1^, which was manually chosen to give the optimal indexing rate. When the number of inter-spot vectors within the network exceeded 20, the corresponding indexing solution was confirmed by initial orientation-matrix refinement (Ginn, Messerschmidt *et al.*, 2015 ▸). At this point the majority of inter-spot vector networks provided a correct indexing solution, as judged by orientation-matrix refinement. When indexing was limited to one lattice solution per image, a lattice was found for 92% of images, a higher proportion of indexed images than the result for rotation-matrix clusters. Enabling multiple lattice indexing (up to three distinct lattices on one image) increased the indexing rate to 151%. Of the 29 images which failed to produce any orientations after indexing, 18 were weak or of low resolution, two showed no evidence of diffraction, one had over 1000 spots (at the extreme of the distribution shown in Fig. 4 ▸) and eight showed no obvious reasons for indexing failure. Another sample of 1380 CPV17 images which extended to 1.84 Å at the edge of the detector demonstrated an indexing rate of 99.3% when only considering single lattices.

**Figure 5:**
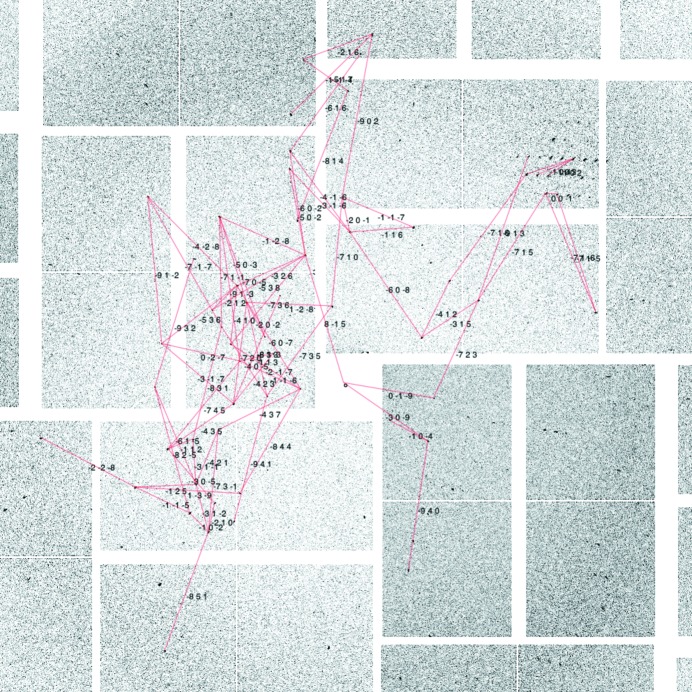
Map of inter-spot vectors within an image of a thermolysin crystal diffraction pattern which contribute to a correct indexing solution. The Miller index translation between the spots is marked beside each vector. The total number of vectors was limited to 80.

Thermolysin crystals were indexed, selecting vectors with lengths of up to 0.1 Å^−1^ also manually chosen to give the best indexing rates. There was a high incidence of multiple lattices in this data set, which made finding inter-spot vector networks more difficult (example in Fig. 5 ▸). However, when searching for multiple lattices an indexing rate of 90% was achieved for this data set.

BEV crystals performed similarly to CPV17 crystals but had to be processed several times with differing combinations of parameters (Table 3 ▸) to achieve the highest indexing rate of 97.6% from a pool of 304 images, despite the spots being much closer together. The greatest distance considered in reciprocal space is 0.06 Å^−1^. The inter-spot vector network is likely to comprise more vectors which span an appreciable distance across the detector, including longer vectors where the errors are proportionately lower.

With respect to myoglobin, 69% of the crystals, which were crystallized in space group *P*2_1_2_1_2_1_ with distinct unit-cell axes, generated an indexing solution using the inter-spot vector-network method using a vector distance tolerance of 5 × 10^−4^ Å^−1^. When searching for multiple lattices, the indexing rate increased to 112%. Inter-spot vectors were considered within a reciprocal-lattice distance of 0.15 Å^−1^. The failed crystals were generally those of lower resolution and had insufficient spots to support indexing with the chosen parameters. Overall, all the images had an average of 148 ± 106 reflections and those which failed to index had a lower average of 80 ± 22 reflections, illustrating the poorer crystal quality of these images.

### 3.4. Tolerance to errors in experimental parameters   

**Figure 6:**
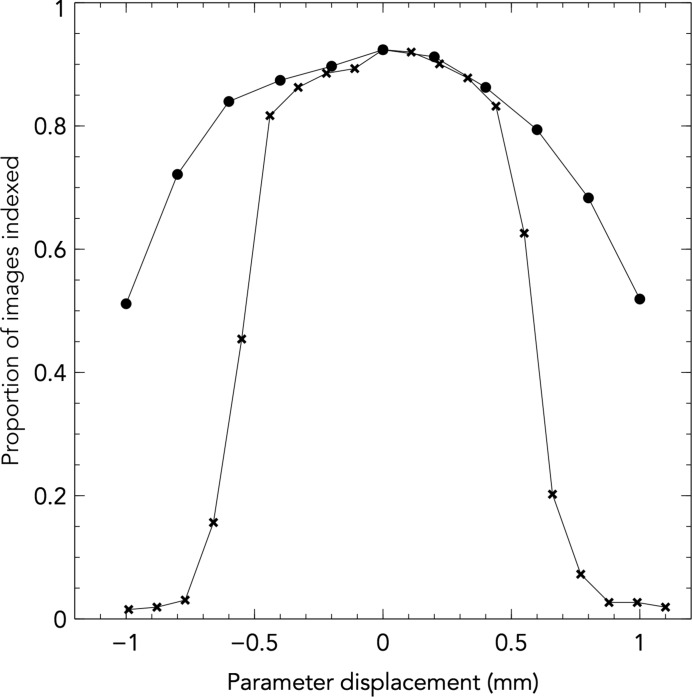
Tolerance of the indexing algorithm to the detector-distance parameter (filled circles) and beam-centre *X* parameter (crosses) are shown. Indexing success decreases significantly after 0.5 mm displacement from the optimal detector distance. The CSPAD detector pixel size is 0.11 mm. Calculated for the CPV17 sample at 101.2 mm detector distance.

To test the robustness of the algorithm, the experimental parameters were altered and the effect on the indexing success rate was observed using CPV17 data. As the detector distance and beam centre are the most likely to be incorrectly measured at an XFEL beamline, these parameters were varied. The detector distance was varied from its true value of 101.2 mm by 1.0 mm in either direction, and similarly for the beam centre *X* position. This algorithm had a good tolerance to experimental errors, and although the indexing rates decreased when the model parameters deviated from their true values, this was in the form of a gradual decay. Indexing success only rapidly decreased after moving approximately 0.5 mm from the optimal value for both detector distance and beam centre (Fig. 6 ▸). For comparison, the shortest vector for this space group would have spanned a minimum of 1.77 mm across the detector if it had been located near the beam centre.

## 4. Discussion and conclusions   

For the reliable indexing of diffraction patterns, experimental parameters such as the direct beam position and crystal-to-detector position need to be known reasonably accurately. Powder patterns aggregated from a large number of diffraction patterns are useful in establishing these parameters. We have found that in cases where only a small number of images are available, or the unit cell is large enough to obscure the low-resolution rings or prevent adequate separation of rings, a pseudo-powder pattern can still be generated by considering the projected three-dimensional vectors between spots on the Ewald sphere. We find that such pseudo-powder patterns are generally useful tools for detecting and correcting significant errors in experimental parameters such as the crystal-to-detector distance, which can still be poorly estimated at XFEL beamlines.

We describe two new approaches to indexing still images such as those derived from serial crystallography. The first approach, termed the rotation-cluster method, is well suited to low-resolution data with a small number of reflections, whereas the second method, using inter-spot vector networks, was found to be more generally robust and particularly suited to the test case in a hexagonal space group. Taken together, the methods presented here have been shown to be highly effective for cubic (CPV17 and BEV), hexagonal (thermolysin) and orthorhombic (myoglobin) space groups, and are likely to be of general utility. In the test cases chosen, the indexing rate was high and the number of lattices indexed ranged from 80 to 150% of the number of images classified as harbouring diffraction.

The large majority of the images which failed to be indexed by the methods described here appeared to originate from weak crystals resulting in diffraction to only low resolution, low XFEL pulse intensity or a combination of these, as judged from the 262-image sample of CPV17. This provides reassurance that the images which failed to index would have provided the least amount of useful information for the data-reduction stage if they had been successfully indexed.

The *TakeTwo* algorithm could be incorporated into other XFEL crystallography software suites as an alternative method. Combining the results from multiple algorithms and removing duplicate solutions should lead to a larger indexed percentage than that obtained using any one algorithm alone. We include the *TakeTwo* algorithm in the *cppxfel* software suite (Ginn *et al.*, 2016 ▸). Potential users may download this software and to do so should visit http://viper.lbl.gov/cctbx.xfel/index.php/Cppxfel. 

## Figures and Tables

**Table 1:** Spot-finding parameters used for *DIALS* spot finding prior to indexing Full parameter definitions are available at the *DIALS* website (http://dials.lbl.gov/). A summary follows. Gain: the ratio of the detector output to the input number of photons per pixel. Global threshold: all pixels less than this value are considered to be background. Minimum spot size: minimum number of pixels required to perform the thresholding operation. Sigma background: number of standard deviations of the coefficient of variation in the local area below which a pixel is classified as background. Sigma strong: number of standard deviations above the mean in the local area above which a pixel will be classified as strong. Low/high-resolution cutoff: lowest/highest resolution considered in spot finding.

Parameter	CPV	BEV	Thermolysin	Myoglobin
Gain	14	14	14	0.1
Global threshold	0	2000	800	0
Minimum spot size	2	2	2	1
Sigma background	6	30	6	6
Sigma strong	3	6	3	3
Low-resolution cutoff (Å)	N/A	27	N/A	15
High-resolution cutoff (Å)	N/A	N/A	N/A	2

**Table 2:** Parameters for each cycle of BEV indexing to achieve the final indexing rate of 97.6% The indexing method used was either the network or the cluster method. Runs were attempted aiming to index a maximum of one lattice per image. Runs were carried out with a constant reciprocal distance tolerance between vectors. Subsequent ‘network’ method runs were run with altered reciprocal distance tolerance and network threshold number values: the reciprocal distance tolerance was tightened with subsequent runs and the network threshold number was relaxed. The requirement for adding inter-spot vectors which included a spot common with the existing vectors was toggled.

Cycle	Indexing method	Reciprocal tolerance	Threshold number (vectors within network)	Common spots required	Indexed images (cumulative %)
1	Network	4 × 10^−4^	22	False	84.0
2	Network	3 × 10^−4^	15	False	93.0
3	Network	3 × 10^−4^	12	True	97.0
4	Cluster	2.5 × 10^−4^	N/A	N/A	97.6

**Table 3:** Crystal symmetry and wavelength/detector distance for the data sets

Data set	Space group	Unit cell (Å)	Wavelength (Å)	Distance (mm)	Total images	Best indexing rate (%)
CPV17	*I*23	106.1 × 106.1 × 106.1	1.46	101.2	262	151.1
CPV17	*I*23	106.1 × 106.1 × 106.1	1.46	91.0	1380	99.3 (single lattice)
BEV	*F*23	437 × 437 × 437	1.31	85.0	304	97.6
Thermolysin	*P*6_1_22	92.9 × 92.9 × 130.4	1.27	175.0	1866	90.1
Myoglobin	*P*2_1_2_1_2_1_	38.0 × 46.9 × 84.5	1.29	68.6	3767	112.2
